# Effectiveness of Consumption of a Combination of Citrus Fruit Flavonoids and Olive Leaf Polyphenols to Reduce Oxidation of Low-Density Lipoprotein in Treatment-Naïve Cardiovascular Risk Subjects: A Randomized Double-Blind Controlled Study

**DOI:** 10.3390/antiox10040589

**Published:** 2021-04-11

**Authors:** Desirée Victoria-Montesinos, María Salud Abellán Ruiz, Antonio J. Luque Rubia, Daniel Guillén Martínez, Silvia Pérez-Piñero, Maravillas Sánchez Macarro, Ana María García-Muñoz, Fernando Cánovas García, Julián Castillo Sánchez, Francisco Javier López-Román

**Affiliations:** 1 Health Sciences Department, Campus de los Jerónimos, Universidad Católica San Antonio de Murcia (UCAM), Carretera de Guadalupe s/n, 30107 Guadalupe, Murcia, Spain; dvictoria@ucam.edu (D.V.-M.); msabellan@ucam.edu (M.S.A.R.); ajluque@ucam.edu (A.J.L.R.); sperez2@ucam.edu (S.P.-P.); msanchez4@ucam.edu (M.S.M.); amgarcia13@ucam.edu (A.M.G.-M.); fcanovas@ucam.edu (F.C.G.); 2 Nursing Department, Campus de los Jerónimos, Universidad Católica San Antonio de Murcia (UCAM), Carretera de Guadalupe s/n, 30107 Guadalupe, Murcia, Spain; dguillen@ucam.edu; 3 Research and Development Department, iff-Murcia Natural Ingredients, Camino Viejo de Pliego s/n, 30820 Alcantarilla, Murcia, Spain; jcsanchez@ucam.edu; 4 Food Technology & Nutrition Department, Campus de los Jerónimos, Universidad Católica San Antonio de Murcia (UCAM), Carretera de Guadalupe s/n, 30107 Guadalupe, Murcia, Spain; 5 Primary Care Research Group, Biomedical Research Institute of Murcia (IMIB-Arrixaca), 30120 El Palmar, Murcia, Spain

**Keywords:** antioxidant, cardiovascular risk, flavonoids, food supplements, oleuropeosides, olive leaf extracts, polyphenols

## Abstract

The aim of the study was to assess whether oral intake of a nutraceutical product (Citrolive™) could determine changes in low-density lipoprotein (LDL) oxidation and other parameters of lipid metabolism and plasma atherogenic capacity. Citrolive™ is a commercial extract obtained from the combination of citrus fruit flavonoids and olive leaf extracts. Twenty-three untreated subjects (69.6% males, 30.4% females, mean age 41.9 ± 9.4 years) with cardiovascular risk factors and a total cholesterol level >200 mg/dL and LDL cholesterol (LDL-C) > 130 mg/dL participated in a 3-month randomized double-blind controlled study. Participants in the intervention group (71.4% males, 28.6% females, mean age 42.7 ± 9.7 years) consumed Citrolive™ (500 mg, two capsules/day), and controls (66.7% males, 33.3% females, mean age 40.6 ± 9.4 years) received a matched placebo. At 3 months, oxidized LDL (ox-LDL) decreased significantly in the intervention group from 93.8 ± 19.1 U/L to 62.8 ± 28.7 U/L (*p* < 0.05), whereas the control group increased from 98.2 ± 23.5 U/L to 105.7 ± 21.9 U/L (*p* = 0.1). Between-group differences were also significant (*p* < 0.05). Similar findings in the ox-LDL/LDL-C ratio were observed. Serum paraoxonase activity (PON1) increased significantly in the intervention group from 64.5 ± 15.6 U/L to 78.7 ± 28.8 U/L (*p* < 0.05) but remained unchanged in controls. Consumption of Citrolive™ for 3 months in treatment-naïve subjects with moderate risk of atherosclerosis was associated with a reduction in oxidized LDL-C and LDL-oxidase/LDL-C ratio as compared to controls.

## 1. Introduction

Atherosclerotic cardiovascular disease (CVD) resulting from the formation of fibrofatty plaques in the arterial wall has spread worldwide, constituting a major public health problem related to the impact of the disease on morbidity and mortality of the population, as well as increasing health care costs around the word. In 2015, more than 17 million people died from CVD, representing 31% of all global deaths [1]. Improvements in cardiovascular health, implementation of preventive measures, and treatment advances may limit CVD mortality, but heart disease and stroke remain a matter of concern due to the epidemic of diabetes and obesity, especially in low- and middle-income countries, and due to the complex interplay between genetic, environmental, and prognostic cardiovascular risk factors [2,3]. Consistent evidence from genetic studies, prospective epidemiologic cohort studies, Mendelian randomization studies, and randomized intervention trials support a consensus statement from the European Atherosclerosis Society Consensus Panel where it has been established that low-density lipoprotein (LDL) is a causal factor in the pathophysiology of atherosclerotic CVD [4]. Infiltration and retention of LDL particles (and apolipoprotein B (apoB), the main structural protein of LDL) in the artery wall is a critically initiating event that sparks an inflammatory response and promotes foam cell formation [5,6]. Oxidative modification converts LDL into atherogenic particles. Uptake and accumulation of oxidatively modified LDL (oxLDL) by macrophages initiates a wide range of bioactivities that may drive development and progression of atherosclerotic lesions [7,8].

Antioxidant interventions using naturally occurring molecules have been a focus of increasing interest as adjuvant nutritional strategies to reduce the risk or progression of cardiovascular disease [9,10]. Dietary supplements and diets rich in antioxidants (vitamin E, vitamin C, vitamin A and carotenoids, polyphenols, flavonoids) aimed to attenuate cell-mediated oxidation of LDL by inhibiting LDL oxidation and cellular lipid peroxidation have shown beneficial effects [11,12,13,14,15]. A combination of citrus flavones and flavanones and olive polyphenols has recently valued for the capability of reducing peroxidative processes, acting as antioxidants, and for the protective effects in experimental models of acute and chronic inflammation [16,17,18,19,20]. Supplementation with a mixture of grapefruit, bitter orange fruits, and olive leaf extracts in healthy volunteers during 8 weeks was associated with improvements in CVD-related factors, including endothelial function, blood pressure, and lipid profile, particularly a reduction in oxidized LDL (ox-LDL) [21].

The present study, conducted in treatment-naïve subjects with moderate CVD risk, aimed to examine the effectiveness of a nutraceutical product with a combination of citrus fruit flavonoids and olive leaf polyphenols on serum levels in ox-LDL, cholesterol, triglycerides, high-density and low-density lipoprotein cholesterol (HDL-C, LDL-C), apolipoproteins A1 and B, and paraoxonase/arylesterase 1 (PON1).

## 2. Materials and Methods

### 2.1. Study Design and Participants

A randomized, double-blind, placebo-controlled study was conducted at the Health Sciences Department of Universidad Católica San Antonio de Murcia (UCAM), in Murcia, Spain. Participants were mainly recruited by advertising the study through social networks and mass media. Inclusion criteria were as follows: Caucasian men and women; age between 18 and 65 years; not belonging to the group of people considered as priorities by the Third Joint Task Force for the prevention of CVD due to high risk [22]; serum cholesterol levels >200 mg/dL and/or LDL-C levels >130 mg/dL without pharmacological treatment; cardiovascular risk according to the Systematic Risk Evaluation (SCORE) risk chart [23] of less than 5% for an ischemic event in a 10-year period; not receiving any pharmacological or nutraceutical treatment for any cardiovascular risk factor (hypertension, diabetes, hyperlipidemia); absence of any treatment affecting weight or appetite; and absence of thyroid gland diseases, heart diseases, renal dysfunction, liver dysfunction, lung diseases, or neurological disorders. Exclusion criteria were as follows: the use of any dietary supplement in the last 3 months, willingness to follow a diet during the study, alcohol abuse or consumption of more than three glasses of wine/beer per day, chronic terminal illness, and ineligibility as judged by the investigators.

The study protocol was approved by the Ethics Committee of Universidad Católica San Antonio de Murcia (code F-PR-AC05-01-05, approval date 23 February 2007) (Murcia, Spain) and was registered in the ClinicalTrials.gov (NCT04330937). Written informed consent was obtained from all participants.

### 2.2. Intervention and Study Variables

Participants were randomly assigned to the intervention group (dietary intervention with the nutritional supplement) or to the control group (supplementation with placebo) using a computer-generated table of random numbers but ensuring homogeneity of the study groups regarding age, sex, assessment of cardiovascular risk, and serum total cholesterol levels. The active product was a commercially available nutritional supplement (Citrolive™, iff-Murcia Natural Ingredients, Site Plant Nutrafur, Alcantarilla, Murcia, Spain) based on the combination of two hydroethanolic extracts, one from bitter orange (Citrus aurantium L.), flavanones (naringin, neohesperidin, neoeriocitrin, and hesperidin) and flavones (luteolin-7-glucoside, apigenin-7-glucoside, diosmetin-7-glucoside, luteolin, and diosmetin), and one hydroethanolic extract from olive leaf (*Olea europaea* L., olive secoiridoids, oleuropein family, hydroxytyrosol, etc.). The quantitative composition and absolute content (% *w/w*, according to the corresponding standards) of the main bioactive ingredients have been previously reported [20].

Citrolive™ and each individual ingredient were classified according to the Global Harmonized System (GHS) into category 5 (unclassified substance or of very low toxicity). Subjects in the intervention group were instructed to take two capsules a day (2 × 500 mg each), 12 h apart, for 90 days. Subjects in the control group received identical-appearing placebo capsules (maltodextrin) and followed the same regimen. All patients were advised to maintain their usual diet and the level of physical activity during the study.

Participants were visited at baseline and at 90 days (final visit). At baseline, written informed consent was obtained, fulfilment of the inclusion criteria was checked, and the study product was provided. Clinical assessments included detailed medical history, comorbidity, physical activity, complete physical examination, and cineanthropometric study based on the model of De Rose and Guimaraes [24] using a scale with a height rod (Seca 220; sensitivity ±5 mm height, ±100 g weight), Holtain skinfold caliper and a Holtain pachymeter, and an inextensible tape measure. All anthropometric measurements were performed in compliance with the International Society of Advancement of Kinanthropometry protocol [25]. Anthropometric variables were as follows: weight, height, body mass index (BMI), waist-to-hip ratio, sum of skinfold thicknesses (subscapular, triceps, suprailiac, abdominal, anterior thigh, medial leg), fat mass (using the Carter, Yuhasz and Faulkner equations for women and men, respectively), skeletal mass (through Von Döbeln equation modified by Rocha), residual mass (through Würch equation), and muscle mass (as the difference between total mass and the sum of fat, skeletal and residual mass). Air displacement plethysmography (BOD POD model 2000A, Life Measurements Instruments, Concord, CA, USA) was used to measure body fat. Participants also completed a 3-day food record including 2 weekdays and either a Saturday or Sunday. All these variables were recorded at the end of the study and at the final visit at 90 days.

Venous blood samples were taken after 12 h of fasting at each of the visits (baseline and 90-day visit). Laboratory studies included blood cell count, cholesterol, triglycerides, LDL-C, HDL-C, glucose, creatinine, alanine aminotransferase (ALT), aspartate aminotransferase (AST), and gamma-glutamyl transpeptidase (GGT) using the clinical chemistry analyzer ILAB 600 (Instrumentation Laboratory). Serum apolipoproteins A1 (apo A1) and B (apo B) were determined using an immunoturbidimetric assay based on the measurement of immunoprecipitation at a wavelength of 340 nm as described by Riepponen et al. [26]. Oxidized LDL (ox-LDL) was measured by Human Oxidized LDL ELISA kit (Elabscience Biotechnology Inc., Houston, TX, USA), and paraoxonase/arylesterase 1 (PON1) by a spectrophotometric technique according to the method described by Ferré et al. [27].

### 2.3. Statistical Analysis

The sample size was calculated according to the ox-LDL as the main variable of the study. Considering a standard deviation of ox-LDL levels of 4 U/L reported in a similar population [28], for a precision of 4 U/L with an alpha risk of 5% and statistical power of 80%, 13 subjects in each group were needed, increasing to 15 subjects per group assuming a 15% loss to follow-up.

The per-protocol (PP) data set of all participants who completed the 90-day study period was analyzed. Categorical variables are expressed as frequencies and percentages and continuous variables as mean and ± standard deviation (SD). Data analysis included the chi-square (χ^2^) test or the Fisher’s exact test for comparison of categorical variables between the study groups, and the analysis of variance (ANOVA) for repeated measures, with time (baseline and final) as the within-subject factor and intervention (active nutritional supplement and placebo) as between-subject factor. Statistical significance was set at *p* < 0.05. SPSS version 21.0 (IMB Corp., Armonk, NY, USA) was used for statistical analysis.

## 3. Results

Of a total of 57 eligible subjects, 27 were excluded because they did not meet the inclusion criteria (*n* = 19) or declined to participate (*n* = 8). Of the 19 subjects who did not meet the inclusion criteria, 15 were receiving antihypertensive and/or antidiabetic treatment, and 4 had a cardiovascular risk percentage greater than 5% according to the Systematic Risk Evaluation (SCORE) risk chart.

**Figure 1:**
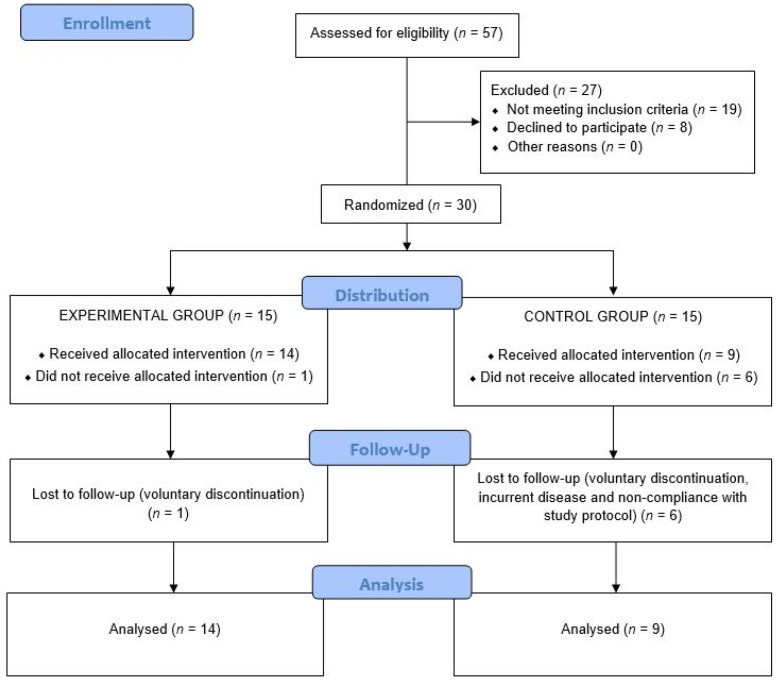
Flow chart of the study population.

The remaining 30 were randomized to the study groups (15 in each group). However, one subject assigned to the intervention group and six subjects assigned to the control group did not complete the study for the following reasons: voluntary discontinuation in four cases, intercurrent disease in two cases, and non-compliance with the study protocol in one case. Therefore, the study population consisted of 23 subjects: 14 in the intervention group and 9 in the placebo group. The flow chart of the study subjects is shown in Figure 1.

A total of 30.4% were women, and the mean age of the study population was 41.9 ± 9.4 years (range 30–59 years). Baseline characteristics are shown in Table 1. There were two patients diagnosed with type 2 diabetes mellitus (both patients aged less than 40 years) and two patients diagnosed with hypertension. None of these four patients received pharmacological treatment, and their diseases were controlled by appropriate diet and physical exercise. Significant differences in the distribution of variables between the study groups were not found.

During the study period, changes in physical activity were not recorded among subjects in the control group, although in the intervention group, percentages of lack of physical activity increased from 35.7% to 42.9%, physical activity <2 h/week from 28.6% to 42.8%, and physical activity 3–5 h/week decreased from 28.6% to 14.3% (*p* < 0.03).

### 3.1. Anthropometric Variables

Results of the cineanthropometric study are shown in Table 2. There were no within-group and between-group significant differences between data registered at the end of the study as compared with baseline in weight, BMI, hip-to-waist ratio, and body fat. However, the sum of skinfold thickness increased in each group, whereas muscle mass showed a decrease, but these changes did not reach statistical significance. Total body fat did not change in any of the study groups throughout the study.

### 3.2. Laboratory Data

Changes in the lipid profile and other laboratory tests in both study groups are shown in Table 3.

**Figure 2:**
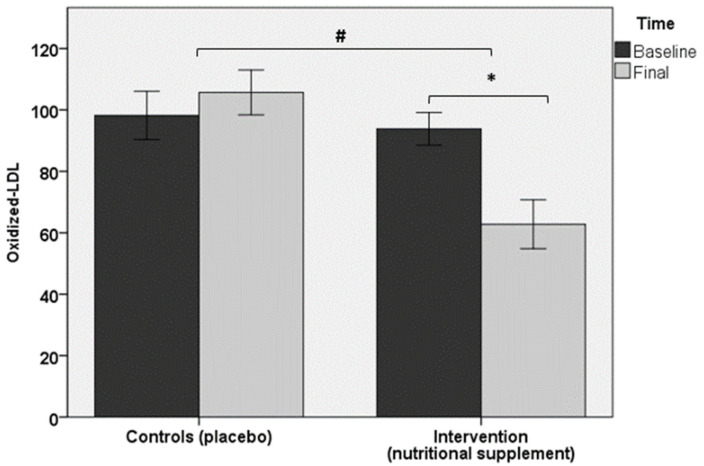
Between-group differences (*p* < 0.05) in the intervention group in ox-LDL values at the end of the study (90 days) as compared with baseline (* asterisk); within-group differences were also significant (*p* < 0.05) (# number sign).

**Figure 3:**
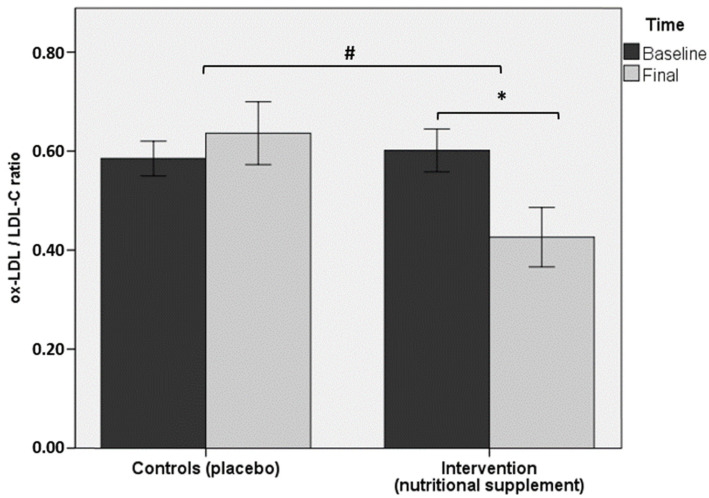
Between-group differences (*p* < 0.05) in the intervention group in ox-LDL/LDL-C ratio at the end of the study (90 days) as compared with baseline (* asterisk); within-group differences were also significant (*p* < 0.05) (# number sign).

Regarding the primary objective of the study, serum levels of ox-LDL showed a statistically significant decrease at the end of the study (62.8 ± 28.7 U/L) as compared with baseline (93.8 ± 19.1 U/L) in the intervention group (*p* < 0.05), but within-group changes in controls were not significant (*p* = 0.1). Between-group differences were also statistically significant (*p* < 0.05) (Figure 2). Similar findings were observed in ox-LDL/LDL-C ratio (Figure 3) and HDL-C/LDL-C ratios. Total cholesterol levels decreased in both study groups, but significant between-group differences were not observed. However, within-group differences in favor of a greater reduction in the intervention group were found (*p* < 0.05). Between-group differences before and after completion of the study in serum levels of triglycerides, LDL-C, HDL-C, apo A1, and apo B were not observed in any of the study groups. PON1 activity increased significantly in the intervention group from 64.5 ± 15.6 U/L at baseline to 78.7 ± 28.8 U/L at 90 days (*p* < 0.05), but changes in the control group were not found.

The use of the nutritional supplements over 90 days did not affect the results of other laboratory tests, including serum hemoglobin, leukocyte count, serum creatinine, and liver enzymes (Table 3). Changes of these parameters in controls throughout the study did not occur.

Finally, as shown in Table 4, variations in nutrient intake during the study period were not observed in any of the study groups.

## 4. Discussion

This randomized, parallel-group, double-blind, and placebo-controlled study showed that nutritional supplementation with a combination of citrus fruit flavonoids and olive leaf polyphenols for 3 months was able to reduce ox-LDL in adults with moderate cardiovascular risk who were treatment-naïve for CVD. Decreases in ox-LDL and ox-LDL/LDL-C at the end of the study as compared with baseline were statistically significant in the intervention group only. Moreover, besides within-group significant differences, between-group comparisons ox-LDL and ox-LDL/LDL-C levels with controls were also statistically significant. These findings have potential clinical relevance given the central role played by ox-LDL in the underlying complex physiopathological mechanisms of formation and progression of the atherosclerotic plaque. It is well known that LDL, the major carrier of cholesterol, accumulates in the intima and stimulates the expression of adhesion molecules and chemoattractants on the surface of endothelial cells, activating the adhesion of circulating monocytes to the endothelium. After adhesion, the monocytes migrate into the intima, differentiate into macrophages, internalize and accumulate the cholesterol in cells, and eventually become foam cells that are characteristic of atherosclerosis [29]. Uptake of ox-LDL by macrophages through scavenger receptors leads to remarkable cholesterol accumulation, converting macrophages to foam cells and promoting the development of atherosclerotic lesions [30].

Plants synthesize an enormous variety of chemicals with powerful antioxidant properties, supporting the notion that increased consumption of fruit and vegetables as well as antioxidant nutrient supplementation reduces the susceptibility of LDL to oxidation, with beneficial effects in respect to various markers of CVD [31,32]. In a 12-week double-blind, placebo-controlled study in patients with established CVD, a high-dose combination of antioxidant nutrients (800 IU of vitamin E, 1 g of vitamin C, 24 mg of beta-carotene) daily reduced susceptibility of LDL to oxidation and may be useful in secondary prevention [32].

Some classes of phytochemical compounds normally present in diets, such as polyphenols, represent particular interesting groups of antioxidants endowed with different mechanisms that can contribute to their capability of reducing peroxidative processes in tissues. On the other hand, flavonoids are a widely distributed group of polyphenolic compounds, characterized by a common benzo-γ-pyrone structure, which has been reported to act as antioxidants in various biological systems [16,17]. Flavonoids are present in a wide variety of edible Mediterranean plants, especially *Citrus* species and olive (*Olea europaea*). Some polyphenols from *Citrus* and *Olea europaea* L. are recently valued as antioxidant compounds and therapeutic agents in the treatment of several vascular disorders. These polyphenolic compounds are responsible for the protective effects in acute and chronic inflammation models in rats [18], are able to scavenge superoxide anion (O2·-) and to depress its production in the cells [33], and may explain some of the protective effects found in cardiovascular disease epidemiological studies [34].

In a previous study, a food supplement based on a combination of grapefruit, bitter orange, and olive extracts administered for 8 weeks improved endothelial function measured by flow-mediated vasodilation, reduced blood pressure and lipid metabolism-related parameters (including ox-LDL), and improved antioxidant and inflammatory status [21]. In the present study, in addition to significant reductions in ox-LDL levels, PON1 increased significantly in the intervention group only. PON1 has antioxidative and athero-protective effects, preventing LDL oxidation. Decreased levels of PON1 are associated with increased risk for CVD [11,35]. Other significant changes in laboratory tests and anthropometric data were not observed. It is of note that there were no variations in nutrient intake during the study period, which further reinforced that changes in ox-LDL, ox-LDL/LDL-C ratio, and PON1 were likely to be associated with the nutritional supplement. It is important, however, to consider that these findings were obtained in a small group of subjects, which is the main limitation of the study, as well as the short duration of the intervention of only 3 months. Therefore, further studies with a larger sample size and duration of consumption of the nutritional supplement are warranted.

## 5. Conclusions

Consumption of a combination of bitter orange fruit flavonoids and olive leaf polyphenols (Citrolive™) for 3 months in treatment-naïve subjects with a moderate risk of atherosclerosis was associated with a reduction in oxidized LDL-C and LDL-oxidase/LDL-C ratio, as well as with an increase in PON1 as compared with the controls.

## Figures and Tables

**Table 1:** Baseline characteristics of the study population.

Variables	Intervention (*n* = 14)	Control (Placebo) (*n* = 9)
Age, years, mean ± SD	42.7 ± 9.7	40.6 ± 9.4
Sex, n (%)		
Males	10 (71.4)	6 (66.7)
Females	4 (28.6)	3 (33.3)
Smoking habit, n (%)		
Current smokers	4 (28.6)	4 (44.4)
Never or ex-smokers	10 (71.4)	5 (55.6)
Comorbidity, n (%)		
Diabetes mellitus	1 (7.1)	1 (11.1)
Hypertension	2 (14.3)	0
Physical activity, n (%)		
None	5 (35.7)	5 (55.6)
<2 h/week	4 (28.6)	1 (11.1)
3–5 h/week	4 (28.6)	3 (33.3)
6–8 h/week	1 (7.1)	0

**Table 2:** Changes of anthropometric variables during the study period.

Variables	Intervention (*n* = 14)		Control (Placebo) (*n* = 9)	
	Baseline Mean ± SD	Final Mean ± SD	Baseline Mean ± SD	Final Mean ± SD
Height, cm	169.9 ± 7.6	169.8 ± 7.5	173.5 ± 9.0	173.6 ± 9.1
Weight, kg	76.9 ± 18.5	76.6 ± 18.3	87.0 ± 15.6	87.2 ± 13.7
Body mass index (BMI), kg/m^2^	26.4 ± 4.5	26.3 ± 4.5	28.9 ± 4.6	28.9 ± 4.1
Waist-to-hip ratio	0.88 ± 0.08	0.89 ± 0.09	0.91 ± 0.07	0.91 ± 0.09
Sum skinfold thicknesses, mm	109.8 ± 25.7	115.9 ± 28.7	137.1 ± 54.2	146.4 ± 52.5
Fat mass, kg	14.3 ± 4.9	15.2 ± 6.3	19.5 ± 8.1	20.4 ± 7.4
Muscle mass, kg	33.1 ± 8.3	32.1 ± 6.8	35.9 ± 6.6	35.0 ± 7.0
Skeletal mass, kg	11.4 ± 1.4	11.2 ± 1.4	12.1 ± 1.7	12.2 ± 1.6
Residual mass, kg	18.1 ± 5.0	18.0 ± 4.9	20.9 ± 4.5	21.0 ± 4.5
Fat, %	18.5 ± 3.7	19.4 ± 3.7	21.8 ± 6.8	23.1 ± 7.4
Muscle, %	43.0 ± 3.0	42.2 ± 2.7	40.8 ± 4.8	39.5 ± 5.5
Skeletal, %	15.2 ± 2.0	15.0 ± 2.2	13.9 ± 1.4	13.9 ± 1.2
Residual, %	23.4 ± 1.4	23.4 ± 1.4	23.6 ± 1.3	23.6 ± 1.3
Body fat, kg	28.7 ± 6.1	28.6 ± 5.3	35.0 ± 8.8	35.0 ± 10.6
Body fat, %	22.0 ± 9.0	22.8 ± 7.6	30.9 ± 10.2	30.9 ± 10.3

**Table 3:** Results of laboratory tests.

Variables	Intervention (*n* = 14)		Control (Placebo) (*n* = 9)	
	Baseline Mean ± SD	Final Mean ± SD	Baseline Mean ± SD	Final Mean ± SD
Total cholesterol, mg/dL	236 ± 27	228 ± 26 *	246 ± 48	241 ± 42
Triglycerides, mg/dL	124 ± 48	108 ± 92	117 ± 46	122 ± 102
LDL-C, mg/dL	159 ± 21	151 ± 17	168 ± 31	170 ± 24
HDL-C, mg/dL	56.5 ± 16.4	57.8 ± 15.6	57.6 ± 16.0	56.8 ± 15.3
HDL-C/LDL-C ratio ^†^	0.36 ± 0.10	0.38 ± 0.10 *	0.36 ± 0.14	0.34 ± 0.11
ox-LDL, U/L ^†^	93.8 ± 19.1	62.8 ± 28.7 *	98.2 ± 23.5	105.7 ± 21.9
ox-LDL/LDL-C ratio ^†^	0.60 ± 0.16	0.43 ± 0.22 *	0.59 ± 0.11	0.64 ± 0.19
Apolipoprotein A1, mg/dL	127.4 ± 25.0	134.2 ± 26.5	126.8 ± 47.9	122.9 ± 37.7
Apolipoprotein B, mg/dL	110.8 ± 13.2	105.6 ± 10.2	123.2 ± 35.4	114.7 ± 28.2
Paraoxonase, U/L	64.5 ± 15.6	78.7 ± 28.8 *	81.6 ± 26.3	80.4 ± 27.4
Hemoglobin, g/dL	14.9 ± 1.4	14.3 ± 1.1	14.6 ± 1.0	14.3 ± 1.0
Leukocyte count, × 10^9^/L	6.1 ± 1.5	5.9 ± 1.6	6.9 ± 1.5	7.0 ± 1.2
Creatinine, mg/dL	0.9 ± 0.2	1.0 ± 0.2	0.9 ± 0.2	1.0 ± 0.2
ALT, U/L	28.7 ± 17.9	20.2 ± 7.6	33.8 ± 16.7	45.1 ± 47.4
AST, U/L	21.9 ± 5.2	18.8 ± 6.8	25.5 ± 7.2	30.6 ± 22.0
GGT, U/L	41.5 ± 46.3	31.4 ± 23.3	34.8 ± 22.7	46.4 ± 40.3

LDL-C: low-density lipoprotein cholesterol; HDL-C: high-density lipoprotein cholesterol; ox-LDL: oxidized LDL; ALT: alanine aminotransferase; AST: aspartate aminotransferase; GGT: gamma-glutamyl transpeptidase. * Indicates statistically significant differences between baseline and end of the study (*p* < 0.05); ^†^ Indicates statistically significant differences when comparing the evolution between groups (*p* < 0.05).

**Table 4:** Result of the 3-day dietary survey at baseline and at the end of the study.

Variables	Intervention (*n* = 15)		Control (PLACEBO) (*n* = 9)	
	Baseline Mean ± SD	Final Mean ± SD	Baseline Mean ± SD	Final Mean ± SD
Energy, kcal/day	2422.42 ± 410.61	2675.05 ± 554.94	3238.89 ± 307.69	3100.26 ± 253.56
Carbohydrates, g/day	247.51 ± 52.69	263.31 ± 60.79	393.40 ± 77.50	359.50 ± 43.98
Proteins, g/day	94.90 ± 25.27	117.08 ± 49.73	125.88 ± 1.39	107.75 ± 6.55
Saturated fatty acids, g/day	26.55 ± 12.43	34.31 ± 11.07	46.80 ± 1.41	60.15 ± 12.94
Monounsaturated fatty acids, g/day	39.66 ± 12.74	56.27 ± 15.96	52.25 ± 3.18	67.70 ± 3.25
Polyunsaturated fatty acids, g/day	15.34 ± 3.94	23.38 ± 20.29	13.70 ± 0.71	18.15 ± 1.48
Cholesterol, mg/day	560.91 ± 286.22	449.11 ± 180.45	562.05 ± 65.83	540 ± 243.81

## Data Availability

No new data were created or analyzed in this study. Data sharing is not applicable to this article.
